# Unmasking the hidden culprit: neurosyphilis mimicking parkinsonism in a middle-aged male

**DOI:** 10.1186/s12883-025-04101-y

**Published:** 2025-03-07

**Authors:** Zhaobo Shi, Yong Sun, Xinsheng Han

**Affiliations:** Department of Neurology, Kaifeng Central Hospital, Kaifeng, 475000 Henan Province China

**Keywords:** Parkinsonism, General paresis, Neurosyphilis

## Abstract

**Background:**

General paresis, a tertiary manifestation of neurosyphilis affecting the brain, is characterized by mental and behavioral disorders, such as attention disorder, cognitive impairment, and personality changes. But parkinsonism is rarely reported in patients with neurosyphilis, let alone general paresis. This study reports a case suffering from both general paresis and parkinsonism.

**Case presentation:**

A 50-year-old man was initially misdiagnosed with “alcohol-related psychiatric and behavioral disorders” due to the onset of psychiatric symptoms following alcohol abstinence. The excessive administration of psychotropic medications, attributed to their limited efficacy, was identified as the primary cause of his subsequent extrapyramidal symptoms, including tremor and bradykinesia. However, treatment with levodopa yielded only marginal effectiveness. Following a comprehensive diagnostic evaluation, which encompassed brain magnetic resonance imaging, syphilis screening, and cerebrospinal fluid analysis, neurosyphilis was ultimately identified as the underlying etiology. Subsequent treatment with aqueous penicillin resulted in a marked improvement in his symptoms.

**Conclusion:**

This case illustrates a rare manifestation of neurosyphilis, specifically parkinsonism. The diagnostic process was complicated by several confounding factors. As neurosyphilis is known as the “great imitator,” capable of mimicking various neuropsychiatric disorders, routine syphilis screening is imperative for patients presenting with mental disorders and parkinsonian symptoms to facilitate early diagnosis and enhance prognosis.

## Background

Neurosyphilis, caused by *Treponema pallidum (T. pallidum)* invading the central nervous system, can affect the brain, meninges, blood vessels, spinal cord, or peripheral nerves [1]. Based on pathological changes, neurosyphilis can be categorized into parenchymatous and meningovascular types. Parenchymatous neurosyphilis, often appearing years post-infection and referred to as general paresis [2], constitutes 57.3% of cases in China [3]. This type is characterized by psychiatric symptoms such as attention disorders, cognitive impairment, and emotional and personality changes. Some patients with neurosyphilis may also present with physical symptoms, including pupillary abnormalities, speech disorders, involuntary facial and hand movements, seizures, and stroke [4, 5]. Instances of neurosyphilis complicated by parkinsonism are exceedingly rare on a global scale. This paper discusses the case of a middle-aged male patient diagnosed with neurosyphilis, who presented with symptoms indicative of general paresis and parkinsonism. Notably, despite a two-year period of misdiagnosis that exacerbated his condition, the patient achieved near-complete recovery following appropriate anti-syphilis treatment.

### Case presentation

At admission, a 50-year-old man complained a two-year history of psychiatric disorders, accompanied by six months of tremor and bradykinesia, and a three-month period of delayed reactions and cognitive impairment. His psychiatric symptoms, which included unrealistic fear, anxiety, restlessness, incoherent speech, insomnia, and visual hallucinations, manifested immediately following alcohol abstinence. Consequently, he was initially misdiagnosed with “alcohol-related psychiatric and behavioral disorders” and was prescribed oral psychotropic medications. The ineffectiveness of these medications prompted an increase in their daily dosage, which was initially suspected to be the cause of subsequent extrapyramidal symptoms. These symptoms included postural and action tremors affecting the upper limbs, bradykinesia, impaired fine motor skills, difficulty initiating gait, a forward-leaning posture with small steps, slow ambulation and turning, and decreased limb coordination. However, adjustments to the psychotropic drug regimen and the addition of levodopa failed to ameliorate his condition, and his cognitive function deteriorated three months prior to admission. The patient had no significant medical history but reported involvement in prostitution a decade earlier. Three years ago, he drank heavily (about 500 g daily) but has been sober for two years.

Clinical examinations of the circulatory, respiratory, and abdominal systems yielded normal results. Neurologically, the patient exhibited mild dysarthria, a masked facial expression, and impaired memory, while maintaining normal emotional responses and self-awareness. Cranial nerve examination revealed no abnormalities. The patient demonstrated increased muscle tone, diminished tendon reflexes, normal limb strength, and an absence of Babinski signs. Although coordination tests posed challenges, they were executed accurately. Both the Romberg sign and the pull test were positive. The patient’s baseline Unified Parkinson’s Disease Rating Scale-III (UPDRS-III) score was 45, and following administration of levodopa and benserazide hydrochloride, an improvement of 11.1% was observed over a four-hour period.

**Fig. 1:**
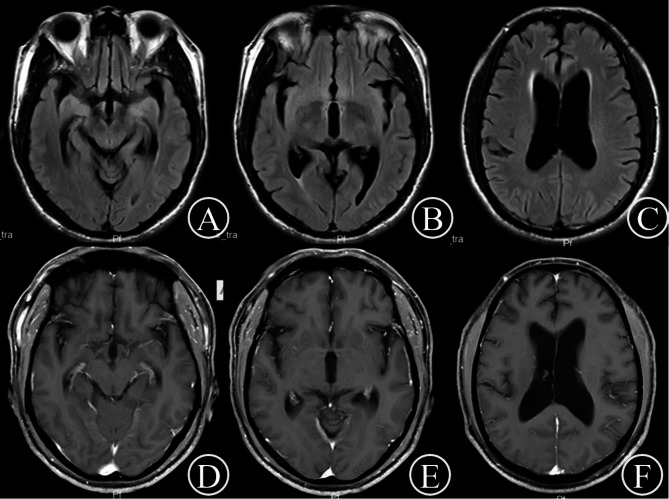
Brain MRI revealed mild brain atrophy in the frontal and temporal lobes and hippocampi and mild supratentorial hydrocephalus (**A**, **B**, **C**); no enhancement was observed after gadolinium injection (**D**, **E**, **F**)

**Fig. 2:**
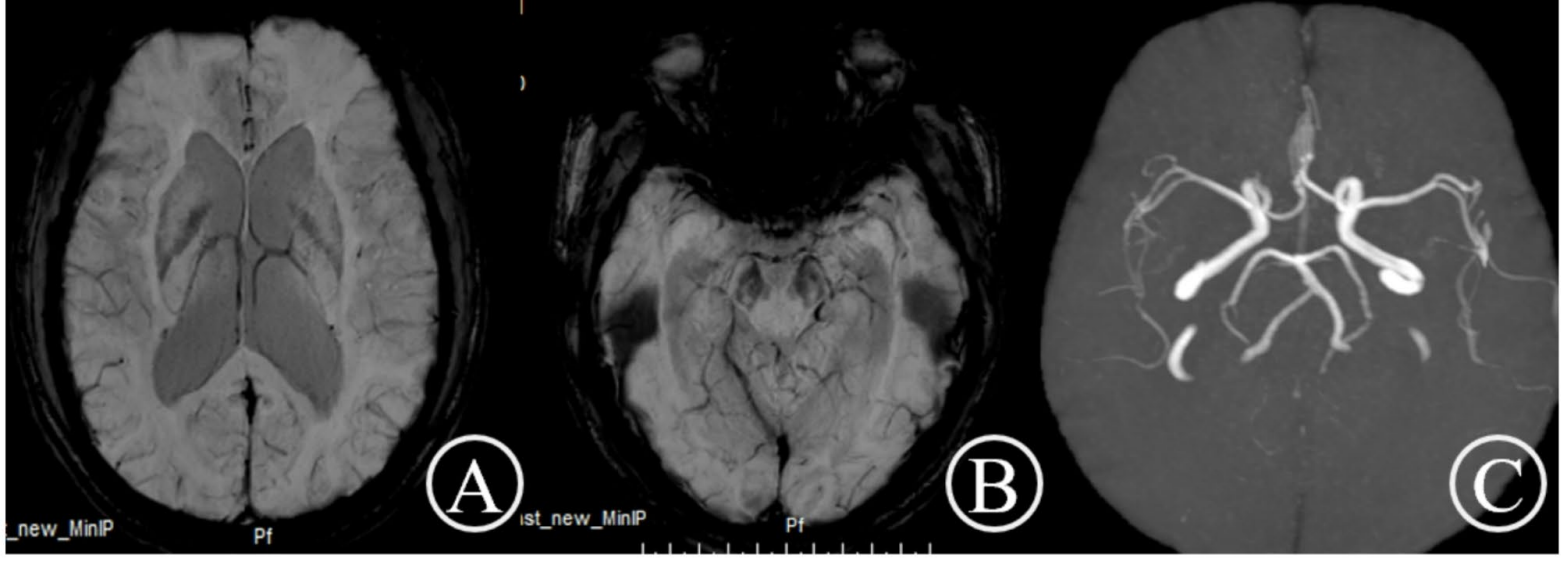
Susceptibility-weighted imaging (**A**, **B**) and magnetic resonance angiography (**C**) revealed no obvious abnormalities

Routine hematological, biochemical, and thyroid function analyses yielded normal results. Infection screening revealed the presence of *T. pallidum* antibodies and a positive rapid plasma reagin (RPR) test with a titer of 1:4. Cognitive assessments using the Montreal Cognitive Assessment (MoCA) and the Mini-Mental State Examination (MMSE) resulted in scores of 13 and 15, respectively. Electroencephalogram findings were unremarkable. Brain magnetic resonance imaging (MRI) demonstrated mild atrophy in the frontal and temporal regions, hippocampal atrophy, mild supratentorial hydrocephalus, and no gadolinium enhancement (Fig. 1). Susceptibility-weighted imaging (SWI) and magnetic resonance angiography (MRA) did not reveal any significant abnormalities (Fig. 2). Cerebrospinal fluid (CSF) analysis showed clear fluid with an opening pressure of 190 mmH2O, an elevated white blood cell (WBC) count of 55.0 × 10^6/L, a positive venereal disease research laboratory (VDRL) test, and an elevated total protein concentration of 0.70 g/L (normal range: 0.15–0.45 g/L). Glucose and chloride concentrations were within normal limits. The concentration of Treponema pallidum antibodies was 42.138 s/co, and the CSF RPR test was positive with a titer of 1:4.

Medications with the potential to induce drug-induced parkinsonism were systematically discontinued, while interventions targeting anxiety, sleep disorders, and cognitive impairment were initiated. Additionally, levodopa and benserazide hydrochloride were administered to alleviate extrapyramidal symptoms. After five days, a slight improvement in the patient’s limb tremor was observed, although psychiatric and cognitive disorders persisted. A diagnosis of late-stage neurosyphilis was confirmed through clinical evaluation and CSF analysis. The patient subsequently received oral prednisone (15 mg twice daily) for three days, followed by intravenous aqueous penicillin (4 million units every four hours) for two weeks, and then benzathine penicillin (2.5 million units weekly) for three weeks. Three months post-treatment, significant improvement was noted in the patient’s psychiatric and Parkinsonian symptoms, along with a slight enhancement in recent memory. Symptoms did not recur following the discontinuation of levodopa and benserazide hydrochloride. Psychotropic medications were gradually tapered over a three-month period, resulting in a marked improvement in cognitive function, as evidenced by a Mini-Mental State Examination (MMSE) score of 24 and a Montreal Cognitive Assessment (MoCA) score of 23. The patient reported no complaints at the 10-month follow-up.

## Discussions and conclusions

In this report, we detail a case of neurosyphilis presenting with parkinsonian symptoms. The patient’s initial psychiatric manifestations emerged immediately following alcohol withdrawal, while extrapyramidal symptoms developed subsequent to the excessive use of psychotropic medications. These factors led to the misattribution of the underlying etiology by the psychiatrist on two occasions. The patient later exhibited cognitive decline, which significantly impacted his daily functioning and led to a consultation with a neurologist. The neurologist successfully identified neurosyphilis as the underlying condition. Treatment with aqueous penicillin resulted in a marked improvement in the patient’s symptoms.

Alcohol-related psychiatric and behavioral disorders encompass withdrawal symptoms following prolonged alcohol consumption, as well as associated personality changes, emotional problems, and psychotic disorders [6]. In this particular case, the patient manifested psychiatric symptoms immediately after alcohol abstinence, leading to an initial misdiagnosis of alcohol-related psychiatric and behavioral disorders. Chronic alcohol intoxication is typically associated with widespread cortical atrophy, thinning of the cortex, and widening of brain sulci and gyri, occasionally accompanied by demyelination of the brain’s white matter [7]. However, these characteristics were not observed in the patient’s brain MRI, as depicted in Fig. 1. Instead, the MRI revealed atrophy of the frontal and temporal lobes, bilateral hippocampal atrophy, and supratentorial hydrocephalus that was atypical for the patient’s age, thus explaining the psychiatric symptoms and cognitive impairment. The brain MRA showed no significant abnormalities, effectively excluding ischemia-related brain atrophy. Notably, neuroimaging findings characteristic of neurosyphilis often include cerebral atrophy, predominantly involving the bilateral frontal and temporal lobes, along with ventricular enlargement [1], consistent with this patient’s MRI findings.

Following a comprehensive evaluation, the patient was ultimately diagnosed with late-stage neurosyphilis, specifically general paresis, in accordance with the 2021 Sexually Transmitted Diseases Treatment Guidelines [4]. In addition to presenting with typical psychiatric and behavioral abnormalities, the patient also demonstrated bradykinesia, bilateral involuntary tremors, and increased muscle tone, aligning with the diagnostic criteria for parkinsonism [8]. The presence of symmetrical extrapyramidal symptoms and only an 11.1% improvement in the UPDRS-III score following a levodopa challenge did not support a diagnosis of Parkinson’s disease. It is noteworthy that certain psychotropic medications prescribed to this patient, including pimozide, magnesium valproate sustained-release tablets, tandospirone capsules, and olanzapine tablets, have the potential to induce parkinsonism [9], which led to an initial misdiagnosis of “drug-induced parkinsonism” by the psychiatrist. However, reducing the psychotropic medications and administering levodopa did not yield improvement. The amelioration of the parkinsonian symptoms following penicillin treatment strongly suggests that the syphilitic infection of the nervous system was the underlying cause of parkinsonism.

Cases of neurosyphilis presenting with parkinsonism are globally rare. In 1953, Neill K. G. reported an atypical case of congenital syphilitic parkinsonism, wherein treatment with penicillin failed to ameliorate the patient’s mask-like facial expression, shuffling gait, and bradykinesia [10]. The prolonged duration of syphilis infection in that case may have contributed to the patient’s poor response to penicillin therapy. In contrast, the parkinsonian symptoms in our patient resolved after two weeks of anti-syphilitic treatment and did not recur during a 10-month follow-up period, despite the cessation of medication. This improvement may be attributed to the relatively brief period during which the the basilar ganglia infected by *T. pallidum.*

The precise mechanism linking neurosyphilis and parkinsonism remains elusive. Some researchers hypothesize that the underlying mechanism may parallel that of viral infections or ischemic damage, leading to disruptions in basilar ganglia function and a reduction in dopamine neurons, receptors, and transmission, or potentially inducing metabolic disorders [11]. Furthermore, Wang et al. observed variations in gut microbiota between patients with neurosyphilis and those with general syphilis, indicating that alterations in gut microbiota among neurosyphilis patients may influence the progression of the disease. It is well-established that gut microbiota plays a significant role in the pathogenesis of Parkinson’s disease, suggesting that changes in gut microbiota may contribute to the onset of parkinsonism in individuals with neurosyphilis [12]. British researchers have reported a case of a neurosyphilis patient who initially presented solely with parkinsonism, experienced significant symptom improvement following syphilis treatment, and subsequently developed primary Parkinson’s disease over a 15-year period [13]. Currently, our patient has remained symptom-free for 10 months and will continue to be monitored throughout his lifetime.

In conclusion, this case illustrates a rare manifestation of neurosyphilis, specifically parkinsonism. The diagnostic process was complicated by several confounding factors. As neurosyphilis is known as the “great imitator,” capable of mimicking various neuropsychiatric disorders, routine syphilis screening is imperative for patients presenting with mental disorders and parkinsonian symptoms to facilitate early diagnosis and enhance prognosis.

## Data Availability

The raw data supporting the conclusions of this article will be made available by the authors, without undue reservation.

## Abbreviations

T. pallidum: Treponema pallidum
UPDRS: Unified Parkinson’s Disease Rating Scale
RPR: rapid plasma reagin
MOCA: Montreal Cognitive Assessment Scale
MMSE: Mini-Mental State Examination
MRI: magnetic resonance imaging
SWI: Susceptibility-Weighted Imaging
MRA: magnetic resonance angiography
CSF: cerebrospinal fluid
